# Pregnancy Complicated by Chronic Pulmonary Aspergillosis

**DOI:** 10.7759/cureus.94851

**Published:** 2025-10-18

**Authors:** Humaira S Malik, Wasib Shah

**Affiliations:** 1 Obstetrics and Gynecology, Dumfries and Galloway Royal Infirmary, Dumfries, GBR; 2 Pulmonology, Dumfries and Galloway Royal Infirmary, Dumfries, GBR

**Keywords:** aspergillus spp, bronchial artery embolization (bae), chronic pulmonary aspergillosis, pregnancy, pulmonary aspergillosis, pulmonary lobectomy

## Abstract

Chronic pulmonary aspergillosis (CPA) is primarily seen in individuals with pre-existing lung pathology like bronchiectasis, cystic fibrosis, chronic obstructive pulmonary disease, or subtle immune compromise. It is rare in pregnancy and poses significant diagnostic and therapeutic challenges. We report a rare case of CPA in a young pregnant woman who was initially diagnosed with bronchiectasis secondary to meningococcal septicaemia in early childhood. She had recurrent haemoptysis during both pregnancies. During her current pregnancy, she presented with worsening respiratory symptoms and was ultimately managed with bronchial artery embolisation. Surgical intervention was deferred until postpartum. A planned caesarean delivery was undertaken at 34 weeks and 2 days due to the risk of massive haemoptysis. Postpartum, she was initiated on antifungal therapy and subsequently underwent definitive treatment with right lower pulmonary lobectomy. This case underscores the importance of appropriate diagnosis and timely management of complex respiratory conditions during pregnancy, particularly the role of a multidisciplinary team.

## Introduction

Pulmonary aspergillosis during pregnancy is a rare but serious condition that poses significant risks to both the mother and the fetus, requiring careful multidisciplinary management. Aspergillosis encompasses a spectrum of diseases caused by fungi of the Aspergillus genus [1], most commonly Aspergillus fumigatus and Aspergillus flavus [2]. Pulmonary involvement may manifest in several forms, including allergic bronchopulmonary aspergillosis, invasive bronchial aspergillosis, chronic pulmonary aspergillosis (CPA), and invasive pulmonary aspergillosis [2].

Among these, CPA represents a long-term, progressive infection that affects lung parenchyma, typically occurring in individuals with pre-existing cavitary or structural lung abnormalities or subtle immune dysfunction [3,4]. However, CPA often presents insidiously, with clinical and radiological features that overlap with those of underlying lung disease, leading to frequent misdiagnosis or delayed recognition [5]. Such diagnostic delays, coupled with suboptimal treatment, contribute substantially to morbidity and mortality [5]. Diagnosis generally relies on characteristic radiographic findings and elevated serum Aspergillus-specific antibody titres [6].

From a clinical perspective, recognising CPA in pregnancy is particularly important, as management decisions must balance maternal safety with fetal well-being. Surgical resection of aspergilloma, when feasible, remains a definitive treatment option in patients with preserved pulmonary function [7].

We present a rare case of CPA in a young pregnant woman, initially misdiagnosed as bronchiectasis secondary to childhood meningococcal septicaemia, where characteristic radiological features (air crescent sign) were initially overlooked. This case underscores the diagnostic challenges of CPA in pregnancy and highlights the importance of a multidisciplinary approach involving obstetric, respiratory, interventional radiology, and thoracic surgery teams to optimise maternal and fetal outcomes.

## Case presentation

A 19-year-old woman, gravida 2 para 1, presented at 22 weeks of gestation with cough, progressive dyspnoea, bilateral pleuritic chest pain, and recurrent small-volume haemoptysis. She denied constitutional symptoms such as fever, weight loss, or night sweats.

Her past medical history was significant for meningococcal septicaemia at the age of five, complicated by severe pneumonia requiring extracorporeal membrane oxygenation. This illness resulted in bronchiectasis and the development of a persistent cyst at the right lung base. Since then, she had experienced recurrent chest infections and was managed with prophylactic antibiotics.

During her previous pregnancy, one year earlier, she developed dyspnoea and haemoptysis, initially attributed to bronchiectasis. A planned caesarean delivery was undertaken due to worsening respiratory symptoms. Postpartum imaging subsequently demonstrated a right lower lobe aspergilloma, confirmed by elevated Aspergillus fumigatus-specific IgG levels and characteristic findings on computed tomography (CT).

In her current pregnancy, respiratory symptoms persisted and were further complicated by gestational diabetes mellitus, diagnosed at 25 weeks of gestation and managed with metformin and continuous glucose monitoring using a Libre device. Her booking body mass index (BMI) was 36.9. She was a non-smoker with no known drug allergies. There were no symptoms to suggest an underlying rheumatological disorder. Routine antenatal investigations, including dating and anomaly scans, as well as subsequent fetal growth assessments, were all unremarkable. Despite this, she continued to experience recurrent chest infections and haemoptysis, which were managed with antibiotics. Tranexamic acid was prescribed for use as required.

**Figure 1:**
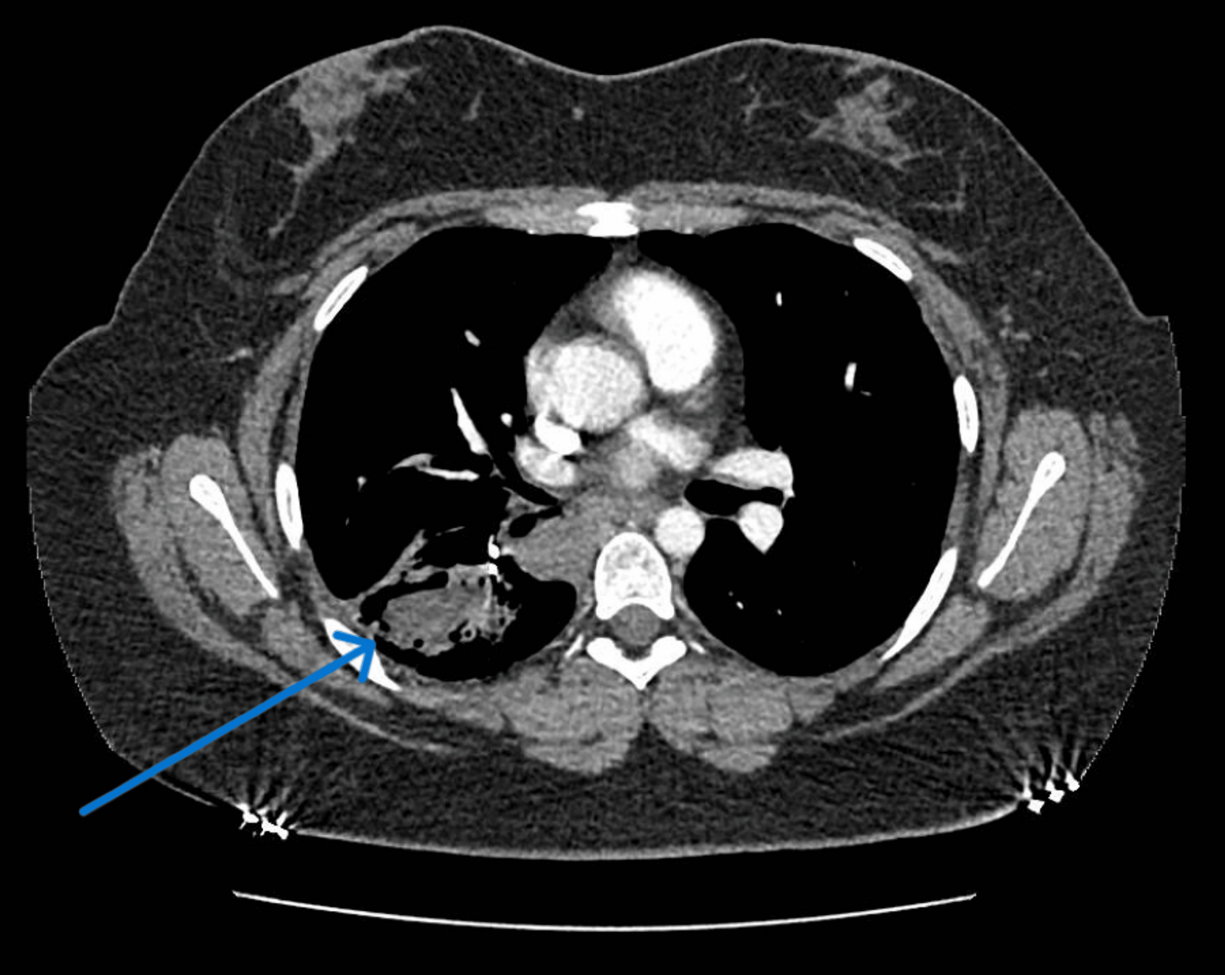
Computed tomography (CT) thorax showing a small fungus ball with an air crescent sign in the right lower lobe.

On examination, her blood pressure was 130/80 mmHg, heart rate 100 bpm, and oxygen saturation 99%. Chest auscultation revealed right-sided crackles with a mild wheeze. Chest radiography demonstrated progressive right lower lobe opacification consistent with aspergilloma. Serological testing for human immunodeficiency virus (HIV) was negative and total immunoglobulin E (IgE)was also measured. Computed tomography (CT) excluded pulmonary embolism but revealed an elevated right hemidiaphragm and areas of consolidation, with a possible air crescent sign suggestive of aspergilloma (Figure 1). A cystic lesion was also noted in the right lower lobe.

Her Aspergillus fumigatus-specific IgG level was elevated at 190, confirming the diagnosis of pulmonary aspergilloma.

Given the recurrent haemoptysis and the associated risk of massive bleeding, a multidisciplinary team (MDT) was convened, comprising specialists in obstetrics, respiratory medicine, infectious diseases, thoracic surgery, and interventional radiology. The thoracic team recommended conservative management of the aspergilloma during pregnancy, with surgical intervention deferred until after delivery unless she developed life-threatening haemoptysis of ≥200 mL. In such an event, the plan was to perform a repeat computed tomography pulmonary angiogram, consult with interventional radiology for possible embolisation, and consider emergency right lower lobectomy if required. Antifungal therapy was also deferred during pregnancy unless massive haemoptysis occurred, with a view to commencing treatment postpartum alongside referral to thoracic surgery for definitive management.

At 29 weeks of gestation, she underwent successful bronchial artery embolisation (BAE) for control of haemoptysis. Although initially effective, she re-presented with recurrent bleeding. Following administration of antenatal corticosteroids to promote fetal lung maturity, a planned caesarean section was performed at 34+2 weeks, resulting in the delivery of a healthy infant. She was discharged on postnatal day six, though she continued to experience small-volume haemoptysis.

At three months postpartum, she remained symptomatic. A repeat CT thorax was arranged to evaluate interval disease progression. Antifungal therapy with voriconazole was initiated after baseline investigations, including electrocardiography (to exclude QTc prolongation) and liver function testing, were normal. She tolerated treatment well but continued to have haemoptysis and a persistent right lower lobe aspergilloma. Following MDT review, she was accepted for surgical intervention. Pulmonary function testing, repeat electrocardiography, and liver function testing were again within normal limits.

She subsequently underwent right thoracotomy and right lower lobectomy. Postoperatively, she made a good recovery and has since remained well, with no recurrence of haemoptysis or chest infections.

## Discussion

Overview and clinical significance

This case highlights the rare intersection of CPA and pregnancy. Infection usually occurs through inhalation of Aspergillus spores from environmental sources such as decaying organic matter, although traumatic inoculation has also been described [3]. CPA most often develops in individuals with underlying cavitary lung disease, such as tuberculosis, sarcoidosis, or bronchiectasis [2-4]. CPA is distinguished from acute and subacute invasive pulmonary aspergillosis by the persistence of symptoms for longer than three months [3].

Underlying conditions and predisposing factors

A published study reported that, after excluding pulmonary tuberculosis and non-tuberculosis mycobacteria, the most common underlying condition was bronchiectasis, followed by chronic obstructive pulmonary disease [5]. Similarly, in our case report, bronchiectasis was also observed as an underlying condition. Interestingly, rare cases have been described in which pleuropulmonary aspergillosis occurred in pregnant women, without any prior lung disease or thoracic surgery [5,8].

Clinical and radiological features

The most common presenting symptoms of CPA include cough, sputum production, and fever, while chest pain and haemoptysis occur less frequently [5]. In our case, the patient presented with cough, breathlessness, and chest pain, highlighting the variable spectrum of disease manifestation. Aspergilloma develops when Aspergillus colonises pre-existing cavities, forming a fungal ball composed of hyphae, inflammatory cells, and fibrin [6]. Radiologically, CPA is classically characterised by a cavity containing a rounded intracavitary mass, often outlined by air, producing the typical “air crescent” or “fungal ball” sign [3]. Aspergillus lung disease presents diagnostic and therapeutic challenges, typically requiring multidisciplinary or subspecialty involvement [2,3].

Diagnosis and laboratory confirmation

The diagnosis of CPA requires a combination of characteristic clinical and radiological findings together with evidence of Aspergillus infection, either by culture of Aspergillus species or by demonstration of elevated Aspergillus-specific antibodies [6]. The presence of high Aspergillus antibody titres, observed in over 90% of patients, is fundamental for establishing a CPA diagnosis [3,6]. In this patient, diagnosis was established on the basis of persistent respiratory symptoms, radiological findings (fungal ball and air crescent sign) and elevated serum Aspergillus IgG.

Complications and management of haemoptysis

Massive haemoptysis is a recognised but potentially life-threatening complication of pulmonary aspergillosis [2]. BAE provides effective short-term control of haemoptysis, but its long-term effectiveness in CPA remains unclear [9]. In our case, BAE effectively controlled haemoptysis, stabilised the patient, and prevented acute complications. A previously reported case of invasive pulmonary aspergillosis with tuberculosis in pregnancy described recurrent haemoptysis necessitating embolisation, emergency caesarean section, and lobectomy [10].

Therapeutic challenges in pregnancy

Management of CPA during pregnancy is particularly challenging due to concerns regarding antifungal safety. Azoles, such as voriconazole, are classified by the U.S. Food and Drug Administration as Category D (fetal risk, but potential benefit may outweigh risk in certain cases) [5]. Although isolated reports of voriconazole use during pregnancy exist [4,11], antifungal therapy was deferred in our case to minimise fetal risk. Early delivery at 34+2 weeks was undertaken due to ongoing haemoptysis, balancing maternal safety with adequate fetal maturation.

Definitive management and surgical outcomes

Therapeutic options for CPA depend on symptomatology, severity of underlying lung disease, and overall patient condition. Surgical resection in specialised centres offers the best potential for complete eradication of disease [12], as demonstrated in this case. Surgical treatment involves resection of both the cavity and aspergilloma, effectively curing the disease and preventing recurrence [6,13]. However, intraoperative spillage of Aspergillus into the pleural space is a known risk, warranting perioperative antifungal coverage [14]. In this case, antifungal therapy was initiated postpartum, followed by right lower lobectomy, resulting in good clinical recovery. This approach aligns with published evidence supporting surgery in well-selected CPA patients with localised disease and preserved pulmonary function, especially when the risk of massive haemoptysis is high [15,16].

## Conclusions

This case illustrates the rare occurrence of CPA complicating pregnancy and highlights the importance of a multidisciplinary approach to balance maternal and fetal outcomes. CPA should be considered in pregnant women with persistent respiratory symptoms and haemoptysis, especially in those with pre-existing structural lung disease. Diagnosis requires integration of clinical history, radiology, and serology. Management during pregnancy is challenging due to limited antifungal safety data and the risk of massive haemoptysis; close monitoring, timely delivery, and definitive postpartum surgical intervention can optimise outcomes. 
